# Institutional Review Boards’ Use and Understanding of Certificates of Confidentiality

**DOI:** 10.1371/journal.pone.0044050

**Published:** 2012-09-04

**Authors:** Laura M. Beskow, Devon K. Check, Emily E. Namey, Lauren A. Dame, Li Lin, Alexandra Cooper, Kevin P. Weinfurt, Leslie E. Wolf

**Affiliations:** 1 Institute for Genome Sciences and Policy, Duke University, Durham, North Carolina, United States of America; 2 Duke Clinical Research Institute, Duke University School of Medicine, Durham, North Carolina, United States of America; 3 Social Science Research Institute, Duke University, Durham, North Carolina, United States of America; 4 Center for Law, Health & Society, Georgia State University College of Law, Atlanta, Georgia, United States of America; University of Pennsylvania, United States of America

## Abstract

Certificates of Confidentiality, issued by agencies of the U.S. government, are regarded as an important tool for meeting ethical and legal obligations to safeguard research participants’ privacy and confidentiality. By shielding against forced disclosure of identifying data, Certificates are intended to facilitate research on sensitive topics critical to the public’s health. Although Certificates are potentially applicable to an extensive array of research, their full legal effect is unclear, and little is known about stakeholders’ views of the protections they provide. To begin addressing this challenge, we conducted a national survey of institutional review board (IRB) chairs, followed by telephone interviews with selected chairs, to learn more about their familiarity with and opinions about Certificates; their institutions’ use of Certificates; policies and practices concerning when Certificates are required or recommended; and the role Certificates play in assessments of research risk. Overall, our results suggest uncertainty about Certificates among IRB chairs. On most objective knowledge questions, most respondents chose the incorrect answer or ‘unsure’. Among chairs who reported more familiarity with Certificates, composite opinion scores calculated based on five survey questions were evenly distributed among positive, neutral/middle, and negative views. Further, respondents expressed a variety of ideas about the appropriate use of Certificates, what they are intended to protect, and their effect on research risk. Nevertheless, chairs who participated in our study commonly viewed Certificates as a potentially valuable tool, frequently describing them as an ‘extra layer’ of protection. These findings lead to several practical observations concerning the need for more stakeholder education about Certificates, consideration of Certificates for a broader range of studies, the importance of remaining vigilant and using all tools available to protect participants’ confidentiality, and the need for further empirical investigation of Certificates’ effect on researchers and research participants.

## Introduction

Researchers and institutions are ethically and legally obligated to safeguard research participants’ privacy and the confidentiality of their data. Indeed, the success of the research enterprise depends on the public’s confidence that private information will be vigorously protected. Certificates of Confidentiality are regarded as an important tool for meeting this expectation. According to federal law [1], researchers who obtain a Certificate may not be compelled in any federal, state, or local civil, criminal, or other legal proceeding to disclose the names or other identifying characteristics of research participants.

Certificates are issued by the National Institutes of Health (NIH) and other units of the U.S. Department of Health and Human Services for studies collecting sensitive information. By shielding against forced disclosure of identifying data, Certificates are intended to facilitate research by reassuring prospective participants about the security of their information and thus allow research to proceed on sensitive topics critical to the public’s health. In March 2002, NIH announced a new policy encouraging broader use of Certificates and establishing a Web-based “kiosk” as a central location for information about Certificates [2]. Their use has recently been promoted in the context of biobanking [3] and large-scale data sharing [4], and NIH currently issues approximately 1000 new Certificates each year [5].

Although Certificates are potentially applicable to an extensive array of research and are commonly believed to offer “nearly absolute privacy protection” [6], there is little evidence concerning the extent and limitations of the protection they provide. A North Carolina Court of Appeals case suggests that the full legal effect of a Certificate is unclear [7]. Empirical data are needed concerning when, why, and how Certificates are used, including stakeholders’ understanding of the protection they provide.

To begin addressing this challenge, we conducted a national survey of institutional review board (IRB) chairs, followed by telephone interviews with selected chairs, to learn more about their familiarity with and opinions about Certificates; their institutions’ use of Certificates; policies and practices concerning when Certificates are required or recommended; and the role Certificates play in assessments of research risk.

## Methods

### Ethics Statement

The Duke University Health System IRB determined that this study was exempt under 45 CFR 46.101(b)(2) and the Georgia State University IRB approved it under expedited review. Prospective participants were provided IRB-approved information about the study and their willingness to complete the survey as their agreement to participate.

### Survey Sample Assembly

We searched NIH RePORTER [8] for new research projects awarded in 2000–2010 by agencies of the federal government most likely to have funded research on sensitive topics: National Institute on Alcohol Abuse and Alcoholism, National Institute on Drug Abuse, National Institute of Mental Health, National Institute of Allergy and Infectious Diseases, Centers for Disease Control & Prevention, and Substance Abuse and Mental Health Services Administration. This search resulted in a list of 1029 uniquely-named institutions in the U.S. that had received funding from these agencies, from which we removed those (n = 61) that were unlikely to have conducted human subjects research involving sensitive data (*e.g.,* institutions dedicated to wildlife or agriculture, professional societies).

We attempted to match each remaining institution (n = 968) to an IRB Organization (IORG) registered in the U.S. using a comprehensive roster obtained from the Office for Human Research Protections (OHRP). For 352 of the institutions, we were unable to identify a matching active IORG. The remaining 616 institutions mapped to 573 IORGs. This sample included 133 of the 136 U.S.-accredited medical schools and 45 of the 49 accredited schools of public health.

Lastly, we selected one chair to whom we could direct our survey at IORGs (n = 98) that operated multiple IRBs. We chose the chair of the socio-behavioral IRB when possible using information from OHRP’s roster (n = 12); otherwise, we chose the chair of the first IRB listed. Survey communications to all prospective participants included the statement, “If you are an IRB chair but would prefer to recommend another chair at your institution who has more experience with Certificates, please let us know.”

### Survey Instrument Development

We drafted the survey instrument based on our knowledge of the issues and literature concerning Certificates [6], [9], [10], [11], [12], [13], [14], [15], [16], [17], [18], [19], as well as the laws, policies, and guidance described on NIH’s Certificates of Confidentiality Kiosk [2]. We revised the instrument through iterative rounds of discussion among our research team, comments from our Expert Advisory Group (see Acknowledgements), and feedback from cognitive pre-testing with four IRB leaders at three major academic institutions. The final instrument (available upon request) consisted of 40 questions, primarily multiple choice and 5-point scale items, and took approximately 20 minutes to complete.

### Survey Implementation and Analysis

We implemented the survey on the Web in January 2011 using Checkbox Survey Software; we did not offer an incentive for participation. Responses were downloaded from Checkbox for analysis using SAS version 9.2.

To get an overall sense of chairs’ views of Certificates, we computed a composite opinion score for each respondent based on five survey questions. These questions sought input on the extent to which Certificates achieve their intended purposes, with responses given on a 5-point scale ranging from “strongly disagree” to “strongly agree.” After reverse-coding negatively worded items, we summed the responses over the five questions. Thus, the composite opinion score could range from 5 to 25, with higher scores indicating a more favorable view of Certificates.

The survey also included six questions to assess respondents’ objective knowledge of Certificates. These questions were based on factual statements available on the NIH Kiosk, with the “correct” answer being the one reflecting NIH’s information. We computed a knowledge score for each respondent based on the proportion of these questions answered correctly.

As exploratory analyses, we compared key findings by years as IRB chair (<5 years versus 5+ years), experience with legal demands for research data (yes versus no), familiarity with Certificates (more familiar versus less familiar), composite opinion score (low [<15] versus middle [15]-[17] versus high [18+]), and knowledge score (<50% correct versus 50%+ correct). The results of these analyses are described only generally because we had limited statistical power to detect differences depending on the factor and survey question; details can be found in Appendix S1.

### Follow-Up Interviews

To explore survey responses in more depth, we conducted 21 follow-up interviews with selected IRB chairs. Our goal was to gain further insights from those whose responses indicated a particularly positive or negative view of Certificates, as well as those whose opinions may have been influenced by experience with legal demands for research data. Thus, we began with a list of all respondents who indicated on the survey willingness to be contacted about a follow-up interview (n = 103). Those who were willing tended to have more years’ experience as IRB chair, more self-reported familiarity with Certificates, and answered more of the objective knowledge questions correctly (Appendix S1).

We invited those who had reported a legal demand (n = 14) to participate in an interview and 8 accepted. We invited additional interviewees based on their composite opinion scores, oversampling among chairs with high or low, versus middle scores. Our final sample consisted of 7 chairs with a high score (4 of whom had experience with legal demands), 4 with a middle score (2 of whom had experience with legal demands), and 10 with a low score (2 of whom had experience with legal demands).

The content of these semi-structured interviews was informed by our survey results, as well as input from the research team and our Expert Advisory Group. We tailored the interview guide (available upon request) for each interviewee to further explore his/her responses to specific survey items. The interviews were conducted by telephone from May to September 2011. One member of the study team (D.K.C.) conducted all of the interviews, with each interview lasting approximately 45 minutes. Verbal consent was obtained and participants were compensated $100 for their time. With participants’ permission, the interviews were audio-recorded, then professionally transcribed for analysis using NVivo 9.

All transcripts were read, structurally- and content-coded, and reconciled by two research team members (E.E.N. and D.K.C.). Content codes were developed based on four interviews and then modified iteratively to reflect themes that emerged from remaining interviews. Narrative segments presented here (along with participant IDs in parentheses) are exemplary of frequently mentioned ideas unless stated otherwise; additional examples are available in Appendix S2.

## Results

### Survey Respondent Characteristics

Of the 573 chairs in our sample, 30 indicated they were ‘not at all familiar’ with Certificates, and thus were determined ineligible. Of the 543 remaining, 246 (45%) responded to our survey. Most were white, non-Hispanic males, age 50 or older, reported more than 5 years’ experience as IRB chair, and were at an academic institution (Table 1). When asked whether their institution had ever received a legal demand to disclose identifiable research data, 10% said ‘yes’. This proportion is likely an underestimate of the prevalence of such demands; when asked to whom an investigator at their institution would be expected to report a legal demand, only 71% indicated the IRB.

**Table 1:** Survey respondent characteristics (n = 246).

	n	(%)*
Years as IRB Chair (mean = 6.3; range = 1–25)		
<5 years	114	(46)
5+ years	132	(54)
Age		
<50 years	65	(26)
≥50 years	178	(72)
Sex		
Male	144	(59)
Female	96	(39)
Race		
White	226	(92)
Other than white	15	(6)
Hispanic		
Yes	4	(2)
No	234	(95)
Current institution		
Academic institution	200	(81)
Non-academic research institute	20	(8)
Non-academic hospital/healthcare setting	8	(3)
Other	16	(7)
Institutional experience with legal demand(s) for identifiable research data +		
Yes	25	(10)
No	127	(52)
Unsure	85	(35)
Number of active protocols with a Certificate †		
None	35	(14)
Less than 20	125	(51)
20–100	49	(20)
More than 100	18	(7)
I am unable to estimate even an approximate number	18	(7)

* May not sum to 100% due to missing data.

+ Responses to survey question “Have any studies at your institution ever received a legal demand (e.g., a subpoena, a court order, or other formal request) to disclose identifiable research data?”.

† Responses to survey question “Approximately how many active research protocols at your institution have a Certificate?”.

### Familiarity with Certificates of Confidentiality

**Figure 1:**
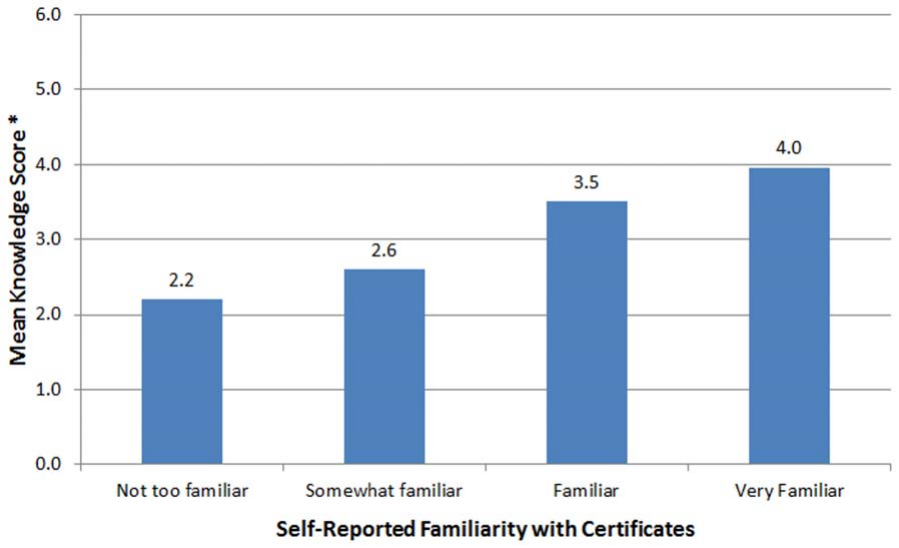
Objective knowledge of Certificates by self-reported familiarity. *Number of correct answers on 6 objective knowledge questions.

Nearly half (45%) of survey respondents characterized themselves as familiar or very familiar with Certificates (Table 2). Those who reported more familiarity tended to have more years’ experience as an IRB chair, answered more of the knowledge questions correctly (Figure 1), and more often reported experience with legal demands for research data.

**Table 2:** Familiarity with Certificates of Confidentiality (n = 246).

	*n*	*(%)*	*n*	*(%)*	*n*	*(%)* *
**Subjective knowledge: How familiar are you with Certificates of Confidentiality? +**						
Less familiar	135	(55)	–	–	–	–
More familiar	111	(45)	–	–	–	–
	Answered		Answered		Answered	
**Objective knowledge: Survey questions**	Correctly †		Incorrectly		‘Unsure’	
The HIPAA Privacy Rule provides the same protections as does a Certificate [FALSE]	193	(78)	9	(4)	33	(13)
A Certificate is granted to the research institution for a particular project, not to the						
Principal Investigator of that project [TRUE]	118	(48)	50	(20)	65	(26)
With a Certificate, a researcher may voluntarily comply with state reporting laws						
(e.g., concerning communicable disease, child abuse), but only when such disclosures						
are specified in the consent document [TRUE]	113	(46)	62	(25)	53	(22)
Research participants are protected only until the expiration date of the Certificate [FALSE]	112	(46)	35	(14)	84	(34)
Even with a Certificate, researchers must release identifiable data to the federal						
government as required for program evaluation or audits of research records [TRUE]	98	(40)	65	(26)	68	(28)
With a Certificate, a researcher may withhold identifiable data, even if the participant						
consents in writing to disclosure [FALSE]	69	(28)	77	(31)	82	(33)
**Objective knowledge: Composite score** ‡						
Answered <50% correctly	106	(45)	–	–	–	–
Answered 50%+ correctly	129	(55)	–	–	–	–

* Percentages may not sum to 100% due to missing data.

+ Less Familiar includes responses ‘not too familiar’ and ‘somewhat familiar’; More Familiar includes responses ‘familiar’ and ‘very familiar’.

† The correct answer (according to factual statements provided on the NIH Kiosk) is shown in [square brackets].

‡ Respondents who answered <3 of the 6 objective knowledge questions shown in THIS table correctly versus those who answered 3+ correctly.

With regard to objective knowledge (Table 2), only slightly more than half (55%) of respondents answered three or more of the six knowledge questions correctly (relative to factual statements on NIH’s Kiosk). Only one question–comparing the protections provided by the HIPAA Privacy Rule to those provided by Certificates–was answered correctly by a majority of chairs. Several questions were answered correctly by nearly half, including items about to whom Certificates are granted, compliance with state reporting laws, and the meaning of a Certificate’s expiration date for participant protections. Less often answered correctly were items about release of data for government audits and the ability to withhold data when participants consent to its disclosure.

### Use of Certificates of Confidentiality

**Figure 2:**
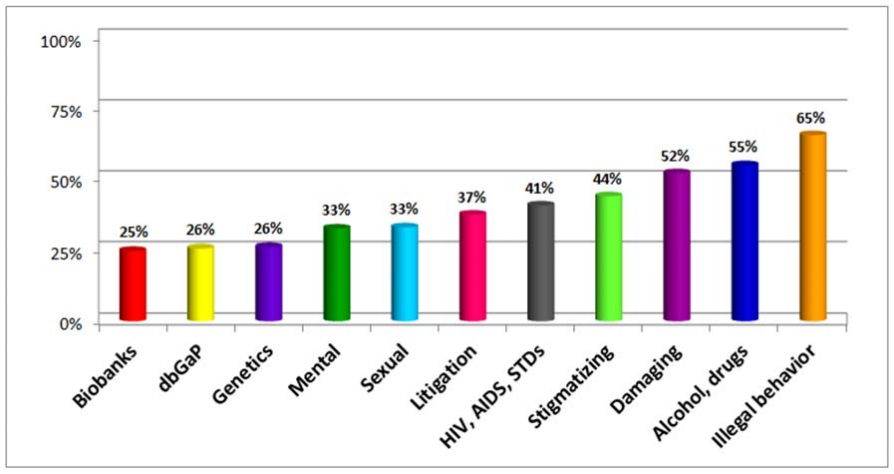
“For which of the following research activities would your IRB typically require or recommend that the investigator apply for a Certificate?”. *Survey respondents were instructed to “Assume in each case that identifiable data will be collected.”. Key: **Biobanks**: Studies that collect and store biospecimens and associated data for future research use. **dbGaP**: Research involving depositing data in centralized repositories for widespread sharing (e.g., placing data from genome-wide association studies into NIH's Database of Genotypes & Phenotypes (dbGaP). **Genetics**: Genetic research. **Mental**: Research on participants' psychological wellbeing or mental health. **Sexual**: Research on participants' sexual attitudes, preferences, or practices. **Litigation**: Research where the topic under study could be the subject of litigation (e.g., breast implants, environmental or occupational exposures). **HIV/AIDS/STDS**: Research on HIV, AIDS, or other STDs. **Stigmatizing**: Research involving information that might lead to social stigmatization or discrimination. **Damaging**: Research that gathers information that could be damaging to a participant's financial standing, employability, or reputation. **Alcohol, drugs**: Research on the use of alcohol, drugs, or other addictive products. **Illegal**: Research that collects information on illegal conduct.

A substantial majority (86%) of survey respondents reported current use of Certificates at their institution (Table 1). We also asked about the types of research for which respondents’ IRBs would typically require or recommend that the investigator apply for a Certificate. We presented a list of research types, based on examples cited on NIH’s Kiosk of research for which a Certificate might be appropriate, and instructed respondents to assume that identifiable data would be collected. The types most commonly selected as those for which a Certificate would be required or recommended (Figure 2) were ‘research that collects information on illegal conduct’ (65%) and ‘research on the use of alcohol, drugs, or other addictive products’ (55%). Research involving genetics, including deposition of data into NIH's Database of Genotypes & Phenotypes (dbGaP) (26%) and biobanking (25%), were those least often selected as a type for which a Certificate might be needed.

With regard to research on illegal activity, our follow-up interviews suggested that some chairs believed Certificates were meant exclusively to protect this kind of information. One interviewee, for example, said Certificates protect behaviors which, if “made public or made available to legal authorities, might result in the research subjects being arrested or at least investigated” (H43803). Other interviewees focused on information about illegal behavior as perhaps not the only, but certainly the most predominant application of Certificates:

“If confidentiality was breached because an attorney subpoenaed your [interview] files, I might get embarrassed, but nothing I’m saying here is illegal. Whereas if I am doing research about cocaine/crack use, much of what people are telling me constitutes illegal criminal activity. If the DA subpoenaed my research files, these people are under real risk. While I won’t get into whether they should or shouldn’t go to jail, they certainly shouldn’t do so as part of agreeing to participate in my research.” (L43632)

With regard to topics other than illegal behavior, several factors emerged during our interviews that chairs mentioned as important considerations when assessing the need for a Certificate:

- *Foreseeable risk of litigation:* “If there wasn’t any risk of subpoena, then we wouldn’t [require a Certificate]. Regardless of the data we wouldn’t insist on the thing. So if there’s no legal issue it’s just not relevant.” (L43559)
- *Realistic threat of serious harm:* “It has to be a serious matter and not just a vague possibility that it would be disclosed.” (H43577)
- *Availability of information elsewhere:* “Anything that would be in your medical record, why would we require a Certificate when [someone] can subpoena the medical record?” (L43632)

As for when a Certificate might be appropriate for genetic research, several interviewees again made reference to illegal activity, e.g., genetic research on “causes of criminal behavior” (H43803) and “genetic data that could link somebody to a crime” (L43699). One chair talked about the confluence of genetic information and the sensitivity of the research topic:

“We have a large group on campus that does research … looking for the overlay between environmental and genetic variables. They ask about quite sensitive activities and some that would be illegal … or [that] wouldn’t look so good if a parent admitted their adolescent was involved in these behaviors. But it’s the overlay between the two–we worry about the mix of genetic markers for a particular racial and/or ethnic group and then what that might do in their lives as individuals, or as a group even. So then we recommend the Certificate.” (MD43786)

Reasons mentioned for not perceiving Certificates as important for genetic research included:

- *Genetic information is not sensitive:* “We do not routinely recommend [Certificates] for genetic or banking studies and find it surprising that some groups do. We feel this overblows the risk of those studies as we are not familiar with legal harm that has come of such data collection. We feel this rather dilutes their meaning.” (L43636)
- *Existing genetic privacy laws:* “We have a genetic privacy law here in [state] that we think covers genetic research specifically.” (HD43857)
- *Accessibility of widely-shared genetic data:* “In the case of repositories that are designed to share the data on a widespread basis, and especially repositories that deal with genome-wide association studies, I think the subject is assuming a very high probability of confidentiality not being able to be protected. So I don’t think we would [recommend a Certificate] in a case like that.” (MD43561)

Chairs’ varying perceptions of appropriate uses of Certificates may be explained in part by their understanding of Certificates’ fundamental purpose. In response to our interview question, “What–to your mind–are Certificates intended to protect?”, answers ranged widely from the confidentiality of all data, to identifiable information, to participants, researchers, institutions, and the research enterprise (Table 3).

**Table 3:** “What–to your mind–are Certificates intended to protect?”.

Theme/Sample Interview Quotes
**All data**
•“It’s intended to protect the confidentiality of the data and to provide the assurance that the data will only be seen by the people…identified in the informed consent. It won’t go anywhere that it’s not supposed to go.” (L43768)
•“The role of the Certificate as I see it is basically to protect confidentiality and to strengthen the researcher's ability to protect any information from being requested by any third party.” (L43838)
•“It protects the research information, which includes the data collected and then any identifiers or other things that are descriptive of the person from being compelled in a legal proceeding from being disclosed.” (L44006)
**Identifying information**
•“It’s specific to legal efforts to obtain identifiable information, either about someone’s participation in a study, or their information that they provided as participants in the study.” (LD43814)
•“The identity of people who reveal information for research purposes.” (LD43588)
**Participants**
•“It’s intended to protect subjects when we’re asking them to disclose behavior that puts them at risk were it known.” (L43632)
•“For instance, litigation in breast implants … the Certificate is not there to protect the company; it’s there to protect the subject. That’s why most people I don’t think <1?h 6pt?>would be thinking of a Certificate in the context of one of those breast implant research studies.” (H43803)
**Researchers**
•“I think they also play a role in protecting the researchers themselves. [Certificates] can be very good in removing a concern that a researcher might have, that there’s a kind of research that somebody might not even be willing to try to do if they weren’t able to obtain a Certificate.” (M43623)
•“We have to remind investigators … from time to time, that the Certificate protects them against being forced to disclose their data. A personal lawsuit by the subject protects the subject against the investigator disclosing the data.” (H44037)
**Institutions**
•In some cases it offers protection for others as well, for an institution, for example, for say a medical practice that is being asked to provide information that the subject has authorized, but may be reluctant to do that without knowing that they have this protection. So in addition to the obvious one of the subject, I think there can be some collateral protection that facilitates research.” (MD43561)
•“I think it’s intended to protect we would like to say in theory the participant, but it’s protecting the university’s ass. [Laughter]. And the funders.” (L44010)
**Research enterprise**
•“And in the end, it protects the research enterprise, because we’d like to be able to say we can promise we’ll keep your information confidential and we can live by that promise.” (HD43839)

### Opinions about Certificates of Confidentiality

We explored chairs’ opinions about Certificates with five survey questions (Table 4) based on the primary objectives Certificates are intended to achieve, including protection of identifiable research data and promotion of participation and truthful disclosure in research on sensitive topics.

**Table 4:** Opinions about Certificates of Confidentiality (n = 246).

		Strongly								Neither Agree								Strongly			
		Disagree				Disagree				nor Disagree				Agree				Agree			
		*n*		*(%)*		*n*		*(%)*		*n*		*(%)*		*n*		*(%)*		*n*		*(%)*	
**Protection of Identifiable Research Data**																					
Certificates provide nearly absolute protection against compelled																					
disclosure of identifiable research data	7		(3)		70		(28)		52		(21)		100		(41)		11		(4)		
The scope of protections the Certificates provide (as described in																					
federal regulations) has been upheld in court cases	7		(3)		25		(10)		117		(48)		80		(33)		5		(2)		
Certificates give researchers a false sense of security	5		(2)		55		(22)		104		(42)		68		(28)		7		(3)		
**Promotion of Research Participation**																					
Certificates are an important tool for facilitating participation in																					
studies involving the collection of sensitive information		6		(2)		21		(9)		50		(20)		139		(57)		21		(9)	
Certificates do not appreciably enhance participants’ willingness																					
to provide valid (truthful) data on sensitive research topics		5		(2)		86		(35)		86		(35)		55		(22)		7		(3)	

* May not sum to 100% due to missing data.

#### Protection of identifiable research data

Our survey included items about (1) the level of protection Certificates afford; (2) the extent to which Certificates’ protections have been tested in court; and (3) whether Certificates give researchers a false sense of security.

Opinions were mixed with regard to the level of protection Certificates afford. Although 45% of chairs agreed with the statement *“Certificates provide nearly absolute protection against compelled disclosure of identifiable research data,”* most either disagreed (31%) or selected ‘neither agree nor disagree’ (21%).

In follow-up interviews, comments illustrating a belief that Certificates provide nearly absolute protection included reference to the researcher being “free of the obligation to deliver data in a lawsuit” (MD43786) and the idea that, with few exceptions, other laws “can’t override the Certificate” (HD44066). Some chairs described a Certificate’s protection as nearly absolute by virtue of it providing a significant deterrent to legal demands for research data:

“The perception among lawyers is if there’s a Certificate, find another way to get information; don’t try a subpoena. So it has a huge deterrent function.” (H44037)

Other interviewees, however, characterized a Certificate’s protections as more of an obstacle that must be dealt with:

“At best [Certificates] throw up an additional barrier that may add to the time and expense required to obtain confidential information. That may encourage some compromise or negotiation between parties, but ultimately I don't think it's enough to prevent the release of confidential information in practice.” (L43559)

Some chairs commented on the limits of Certificates’ protection, for example against “the investigator getting a little loose with the information [or] the individual wanting to tell everybody what they told the investigator” (H44037), or against “seeing a participant walking into a study site [or] disclosure that happens because somebody had the information on a portable hard drive that they left in a coffee shop” (M43623).

Some also noted that the extent of the protections depends on the institution’s willingness to fight disclosure “administratively, legally and politically” (H43816), including the need to “invest money in lawyers to make the Certificate stick” (L43559).

Across all of the interviews, a frequent theme was that of Certificates providing an “extra layer” of protection. In this context, many highlighted the importance of standard confidentiality measures:

“Quite simply, we look at the information collected. We look at how it’s labeled or stored, linked to an individual or not, and who has access to it, where it might be stored, how long it’s stored and that sort of thing. I mean we expect a certain level for all studies. And we sort of put all this together and decide, does this pass muster? Might special protections be needed?” (L43636)

Opinions were also mixed about the extent to which Certificates have been tested in court. In response to the statement *“The scope of protections that Certificates provide (as described in federal regulations) has been upheld in court cases,”* 35% of survey respondents agreed, but the majority either disagreed (13%) or chose ‘neither agree nor disagree’ (48%).

In follow-up interviews, one chair who felt that Certificates had been or would be upheld in a court case explained:

“We have read some information and my final impression has been that the Certificate indeed has protected participants and principal investigators in terms of not making him provide information.” (L43838)

Several interviewees, however, thought that Certificates were largely untested and were noncommittal as to the likely outcome of a court challenge, saying for example, “I don’t think we really know that it will be upheld” (LD43588). Other chairs who thought Certificates had not been adequately tested expressed skepticism:

“There have not been a lot of legal challenges and, therefore, it is really in my mind uncertain the level of protection that [Certificates] actually afford. Obviously, in the unhappy circumstance that one would be successfully challenged, there is a huge con to both the subjects and the researchers of having this disclosure happen.” (L44006)

When discussing whether Certificates have been tested in court, several chairs noted reliance on what they had heard from their institutional legal counsel. Even so, their opinions ranged from confidence that a Certificate would be upheld:

“We have two attorneys with my institution and they’ve both indicated that there’s never been a court case that said a Certificate would not be honored. In other words, it’s never been overturned in a court. So that’s pretty strong evidence that the protection will hold up.” (H43577)

to concern that it would not:

“[In a study involving videotape of driving behavior] there were questions about the extent to which [a Certificate] could protect or allow the researchers to resist the request for research data. It was our understanding from our legal counsel that if there were a legal action based on some driving event and the attorneys for either side were aware that this record existed, the Certificate may not protect the researcher entirely from turning over the video.” (L43768)

Opinions were once again mixed with regard to the statement *“Certificates give researchers a false sense of security,”* with 30% of chairs agreeing, 24% disagreeing, and 42% neither agreeing nor disagreeing.

The responses of some chairs we interviewed appeared to be grounded in their basic assessment of Certificates’ effectiveness. For instance, one chair who felt positively toward Certificates said, “I don’t think they’re getting a false sense of security because [a Certificate] is pretty secure” (H43577), whereas another who was more skeptical stated, “A problem with Certificates [is] this potential false sense of security, if they indeed are not as foolproof as I think most researchers and IRBs perceive them to be” (L44006).

Other interviewees were specifically concerned that having a Certificate could lessen researchers’ focus on other confidentiality protections, referencing for example the possibility of “less stringent procedures, practices and vigilance in the provision of more ‘ordinary’ protective measures” (H43816) or “a temptation not to employ very diligent safeguards of the data” (L43768).

Noting that some researchers may have a false sense of security about their data more generally, one interviewee urged increased efforts to raise awareness about legal risks:

“I remember a case years ago where … everybody got sued. And the researcher was, ‘My data!’ I mean sometimes researchers are incredibly naïve. They said, ‘They can’t, I’m a researcher. I’m protected. I shouldn’t have to release my research data!’ I mean [laughter] you’re kidding me! When the lawyers come after you, you’ll do whatever the court’s telling you to do or go to prison… I think there should be a lot better education of researchers that they’re at risk.” (L43559)

#### Promotion of research participation and truthful disclosure

We included survey items about whether Certificates (1) facilitate participation in sensitive research; and (2) enhance participants’ willingness to provide truthful data.

Survey respondents generally held favorable views toward Certificates as a means to promote research participation. A large majority (65%) agreed with the statement *“Certificates are an important tool for facilitating participation in studies involving the collection of sensitive information.”* Only 11% disagreed and 20% selected ‘neither agree nor disagree’.

In follow-up interviews, many chairs spoke about Certificates as giving much-needed reassurance to prospective participants, providing “people with a level of comfort that enables them to be willing to participate in research in the first place” (M43623) and possibly making it easier to retain subjects because “they felt more comfortable and more reassured about things being held in strict confidence” (H44037).

One chair who was less positive about Certificates’ effect on research participation questioned whether the advantages outweighed the disadvantages, given that “you’ve got to explain what the limitations are to human subjects, and that might confuse [or] mislead them. So it’s really difficult to have strong feelings that the net benefits are there” (L43559).

Opinions were more varied with regard to the effect of Certificates on data quality. Although 37% of chairs disagreed with the statement *“Certificates of Confidentiality do not appreciably enhance participants’ willingness to provide valid (truthful) data on sensitive research topics,”* most either agreed (25%) or selected ‘neither agree nor disagree’ (35%).

In follow-up interviews, chairs who felt that Certificates do enhance participants’ willingness to provide valid data again described them as offering reassurance:

“On research with high risk and sensitivity, participants’ willingness to provide … valid data about themselves is going to be influenced by how confidential they think it’s going to be kept. If you have a Certificate, that not only reminds them that there’s some protection against third party intrusion, but that the whole concept of confidentiality is being taken seriously by the research team.” (H44037)

One interviewee, however, questioned whether Certificates were necessary to obtain valid data, saying that “subjects are often willing to disclose rather surprising things without having to go through all this” (H43577), and others raised concerns about falsely reassuring participants:

“I mean it’s not a cone of silence. It’s not the case that this information is somehow not recorded or nobody knows or nobody could ever find out. I think that can lead to a false sense of security on the part of participants. I think also it can lead to … people being willing to take on risks that they might otherwise not have done.” (M43623)

“The disadvantages are I think [Certificates] may provide a false sense of security… I also think people do not understand that some of the potentially most damaging information that they might provide, for example, things about abuse, we still have state reporting requirements there that are not obviated by a Certificate.” (L43636)

Several chairs emphasized trust as a more important factor in participants’ willingness to provide truthful information:

“I really believe that your relationship with the participant enhances their trust and what they’re going to say. Unfortunately, the more documents people see does not necessarily make them feel any better… Anything more from the government, I don’t think helps that in any way.” (L44010)

“If you don’t trust the man, you ain’t giving him the information with or without the Certificate.” (L43632)

#### Opinions about certificates: a composite view

As described under Methods, we computed a composite opinion score based on the five survey questions described above. The mean and median scores were 16, with 43% scoring between 15 and 17. The remaining chairs were split evenly between those holding a more negative view (with scores ranging from 7–14) and those holding a more positive view (with scores ranging from 18–25). Scores for those who reported more familiarity with Certificates suggested an even distribution among positive (36%), neutral/middle (32%), and negative (32%) views. Among those who answered at least half of the knowledge questions correctly, the largest proportion (44%) were in the middle score group. Nearly half (48%) of those who said they had experience with legal demands for research data were in the high opinion score group.

### The Effect of Certificates on Assessments of Research Risk

Finally, we asked survey respondents to consider a research scenario (Box S1), which we devised to portray sensitive research involving identifiable data. We queried them about the level of risk involved in the study without a Certificate, whether a Certificate was needed, and the level of risk and the protection afforded to participants if a Certificate were obtained.

When asked to categorize the level of risk involved in the study *without* a Certificate, 77% selected ‘greater than minimal risk’. Among these chairs (n = 190), responses varied with regard to the need for a Certificate for the study: 46% of chairs said their IRB’s approval would likely be contingent on the investigator obtaining a Certificate, 31% said the research would likely be approved but a Certificate recommended, 13% said the research would likely be approved without a Certificate, and 9% were unsure.

When asked to categorize the risk involved in the study assuming the investigator *does* obtain a Certificate, there was a notable shift toward lower levels of risk compared to our earlier question. Among chairs who had said the study involved ‘greater than minimal risk’ without a Certificate (n = 190), a significant minority chose ‘no greater than minimal risk’ (24%) or ‘no reasonably foreseeable risk’ (3%) with a Certificate.

In follow-up interviews, we asked chairs to tell us more about how a Certificate affects their IRB’s assessment of research risk. One chair who felt a Certificate could lower the level of risk gave this example:

“We had a study that was clearly minimal risk, but it did ask questions about drug use… So we basically gave [the research team] a choice. They could get a Certificate and have it be expedited, or they could bring it to the full board and see if the board would require it.” (L43699)

Other interviewees said that a Certificate would not change their categorization of the level of risk, but rather that it is a “tool to manage risk” (HD43857) and could make the IRB “more willing to approve [a greater than minimal risk] study” (L43838).

Comments from some of the chairs we interviewed suggested that describing plans to obtain a Certificate early in the IRB review process could affect their risk assessment. Interestingly, in one instance the predicted effect was to mitigate confidentiality concerns:

“It certainly would allow us to possibly consider the project as no more than minimal risk if the investigator has shown how they will protect the confidentiality of the subjects.” (LD43588)

whereas in another, the predicted effect was to heighten confidentiality concerns:

“In some ways, [a Certificate is a] flag that the researcher thinks this is potentially a very problematic study. I feel sort of guilty saying that … this plays a role, but it does. I mean if the researcher says, ‘I think I might need this,’ then it certainly gets very closely scrutinized, which is not to say that other studies do not, but I think it certainly is a signal to us as an IRB.” (M43623)

We concluded our survey questions about the hypothetical scenario by asking chairs about the change in protection provided to participants if the investigator obtained a Certificate, compared to the basic protections described in the scenario (without a Certificate). Among all chairs (n = 246), most (59%) said a Certificate would slightly increase protection; others said it would greatly increase protection (19%), make no difference (8%), or slightly (4%) or greatly (3%) decrease protection.

## Discussion

Overall, our results suggest some degree of uncertainty about Certificates among IRB chairs. On our objective knowledge questions, most chairs chose the incorrect answer or ‘unsure’ rather than the answer reflected on NIH’s Kiosk. Most were in the middle opinion score group, expressing neither a particularly positive nor negative view of Certificates. Higher levels of self-reported familiarity with Certificates tended to move respondents out of the middle opinion score group–some toward a more positive view and others toward a more negative view. Further, respondents expressed a variety of ideas about the appropriate use of Certificates and what they are intended to protect, as well as their role in managing versus reducing risk.

Despite this uncertainty, chairs who participated in our study commonly viewed Certificates as a potentially valuable means for facilitating participation in sensitive research and protecting participants’ privacy and the confidentiality of their data. In the hypothetical research study we presented–despite a range of opinions about the level of risk involved and the need for a Certificate–the effect of obtaining a Certificate was a shift toward lower levels of perceived risk and increased protection for participants. In general, chairs frequently described Certificates as an ‘extra layer’ of protection and emphasized the critical importance of ensuring the implementation of more basic confidentiality measures. These findings lead to several practical observations:

First, our data suggest that IRBs’ understanding of Certificates is lacking and more education for human research protection professionals is needed. IRBs play a key role in identifying studies for which a Certificate may be appropriate and researchers likely look to the IRB for guidance about the use of Certificates; thus, it is essential that they have accurate information. Although IRB personnel may look into specific aspects of Certificates as needed, chairs’ generally poor performance on our objective knowledge questions is troubling given that four of the six questions were directly related to core IRB functions (confidentiality-related aspects of HIPAA, state reporting laws, and government audits, as well as participant consent). In addition, it may indicate that some IRB personnel overestimate what they know about Certificates or are unaware of what they do not know. Indeed, IRB chairs reporting the highest level of familiarity with Certificates still had a mean knowledge score indicating some misunderstanding (Figure 1). Education about Certificates should involve active outreach at multiple levels, including greater promotion of NIH’s recently revamped Kiosk, programs by professional organizations, and incorporation into training and certification of IRBs and human research protection professionals.

Second, IRBs may not be using Certificates for the entire range of studies that are eligible for them. The current statute enables the use of Certificates for biomedical, behavioral, clinical or other types of research collecting sensitive data that, if disclosed, could have adverse consequences for participants or damage their financial standing, employability, insurability, or reputation [2]. Available data suggest that applications for Certificates are rarely denied [11]. Even so, a number of chairs who participated in our study expressed a narrow conception of Certificates as being meant only (or primarily) for studies that collect information about illegal behavior. Certificates should be considered for a broader range of research, and the topics listed on NIH’s Kiosk as examples of research for which a Certificate may be appropriate (summarized in Figure 2) are a good place to start. For instance, it is noteworthy that few of our respondents’ IRBs seemed to consider Certificates important for research involving genetics, despite recommendations to the contrary per NIH’s Kiosk, NCI’s Best Practices for Biorepositories [3], and NIH’s “Points to Consider” for genome-wide association studies [4]. This discrepancy may be attributed, in part, to a perceived lack of sensitivity of genetic data given the Genetic Information Nondiscrimination Act [20] and state genetic privacy laws [21], which prohibit certain kinds of discrimination based on genetic information. However, these are not the only ways genetic information could be used to harm someone.

Of course, given broader consideration of Certificates, one could begin to imagine a reason why virtually any piece of data might be of at least theoretical interest in a legal action, leading to the prospect of requiring or recommending a Certificate for nearly every study. Thus, it is important to take into account tempering factors such as those articulated by our study participants, including reasonably foreseeable risk of litigation, realistic threat of serious harm, and the availability of the sensitive information elsewhere.

Third, the mixed opinions we uncovered about the extent to which Certificates protect identifiable research data may be due, in large part, to true uncertainty in the field rather than misunderstanding or lack of knowledge. There is little guidance from the courts on the scope of protection offered by Certificates because there have been very few published opinions that consider the effect of Certificates. In cases that do exist, the varied and unique factual situations involved make it difficult to generalize to other situations. While it is unclear whether Certificates can provide the absolute protection the statute authorizes in all circumstances (for example, if constitutional rights were at stake in a criminal case [7], [22]), it is difficult at this juncture to know how far their protection extends. Chairs in our study offered alternative views of Certificates–a strong deterrent to legal demands versus an obstacle that may help lead to compromise and negotiation. Under either view, chairs underscored the importance of remaining vigilant and using all tools available to protect participants’ confidentiality.

Finally, many of our respondents felt that Certificates do facilitate research participation by reassuring subjects, but they were less inclined to think a Certificate is necessary to ensure the collection of truthful information. Rather, several commented that participants’ trust in the researcher was a more important factor. In reality, these areas have been little studied [9], [10] and require further empirical investigation.

Our national sampling frame, good response rate, and mixed methods approach are important strengths of this work. However, several factors may limit the interpretation of our results. First, we do not have data about the characteristics of chairs who did not respond to our survey and thus cannot assess potential response bias; in general, the demographic characteristics of our respondents were similar to those found in surveys of IRBs on other topics [23], [24]. Second, our online survey comprised primarily closed-ended questions and we had variable power to detect statistically significant differences; further, we had the resources to conduct only a relatively small number of follow-up interviews. Thus our ability to assess even more nuanced factors that might explain or influence IRB chairs’ opinions about Certificates view was constrained. Third, to keep the survey to a reasonable length, we did not include questions covering every possible issue (e.g., IRB chairs’ view and experiences of the ease or difficulty of obtaining a Certificate). Thus, further research is warranted–among IRB leaders and other institutional officials, as well as researchers and research participants–to facilitate the development of sound policies that promote the appropriate use and understanding of Certificates of Confidentiality.

## Supporting Information

Appendix S1 Comparative Analyses of Key Findings.
(PDF)

Appendix S2 Additional Sample Quotes.
(PDF)

Box S1 Hypothetical Research Scenario.
(PDF)
