# Supplementary material for: Institutional Review Boards’ Use and Understanding of Certificates of Confidentiality
Source: PLoS One. 2012 Sep 4;7(9):e44050. doi: 10.1371/journal.pone.0044050 (PMC3433491; doi:10.1371/journal.pone.0044050)
Supplement: Appendix S1 — Comparative Analyses of Key Findings. (PDF) [file pone.0044050.s001.pdf]

APPENDIX S1: COMPARATIVE ANALYSES OF KEY FINDINGS
(Beskow LM, et al. IRBs' use and understanding of Certificates of Confidentiality)

Q40-Willingness to be contacted about a follow-up interview

Measure	Statistic/ Category	Q40: IS willing to be contacted about an interview (n = 103)	Q40: is NOT willing to be contacted about an interview (n = 143)	p Value#
1. For how many years have you been an IRB chair?	n	103	143	0.08^
	5+	62 (60%)	70 (49%)	
	<5	41 (40%)	73 (51%)	
31-37. Number of correct answers in q31 q32 q34 q35 q36 q37	n	93	142	0.02^
	1) <3	33 (35%)	73 (51%)	
	2) 3+	60 (65%)	69 (49%)	
3. How familiar are you with Certificates of Confidentiality? (Recoded)	n	103	143	0.0004^
	1=More familiar	60 (58%)	51 (36%)	
	2=Less familiar	43 (42%)	92 (64%)	
20-24. Composite opinion score = q20+q22+q23+(6-q21)+(6-q24)	N	95	135	0.64^^^
	1) <15	25 (26%)	37 (27%)	
	2) 15-17	42 (44%)	63 (47%)	
	3) 18+	28 (29%)	35 (26%)	
30. Have any studies at your institution ever received a legal demand (e.g., a subpoena, a court order, or other formal request) to disclose identifiable research data?	N	64	88	0.12^
	No	50 (78%)	77 (88%)	
	Yes	14 (22%)	11 (13%)	

Q3-Familiarity with Certificates

Measure	Statistic/ Category	Q3: 1=More familiar (n = 111)	Q3: 2=Less familiar (n = 135)	p Value#
1. For how many years have you been an IRB chair?	n	111	135	0.007^
	5+	70 (63%)	62 (46%)	
	<5	41 (37%)	73 (54%)	
31-37. Number of correct answers in q31 q32 q34 q35 q36 q37	n	109	126	<0.0001^
	1) <3	24 (22%)	82 (65%)	
	2) 3+	85 (78%)	44 (35%)	
20-24. Composite opinion score = q20+q22+q23+(6-q21)+(6-q24)	n	106	124	0.53^^^
	1) <15	34 (32%)	28 (23%)	
	2) 15-17	34 (32%)	71 (57%)	
	3) 18+	38 (36%)	25 (20%)	
30. Have any studies at your institution ever received a legal demand (e.g., a subpoena, a court order, or other formal request) to disclose identifiable research data?	n	75	77	0.01^
	No	57 (76%)	70 (91%)	
	Yes	18 (24%)	7 (9%)	

Knowledge Score

Measure	Statistic/ Category	Number of correct answers in q31 q32 q34 q35 q36 q37 1) <3 (n = 106)	2) 3+ (n = 129)	p Value#
1. For how many years have you been an IRB chair?	n	106	129	0.02^
	5+	48 (45%)	78 (60%)	
	<5	58 (55%)	51 (40%)	
3. How familiar are you with Certificates of Confidentiality? (Recoded)	n	106	129	<0.0001^
	1=More familiar	24 (23%)	85 (66%)	
	2=Less familiar	82 (77%)	44 (34%)	
20-24. Composite opinion score = q20+q22+q23+(6-q21)+(6-q24)	n	100	124	0.09^^^
	1) <15	31 (31%)	30 (24%)	
	2) 15-17	47 (47%)	54 (44%)	
	3) 18+	22 (22%)	40 (32%)	
30. Have any studies at your institution ever received a legal demand (e.g., a subpoena, a court order, or other formal request) to disclose identifiable research data?	n	67	83	0.61^
	No	57 (85%)	68 (82%)	
	Yes	10 (15%)	15 (18%)	

Composite Opinion Score

Measure	Statistic/ Category	Composite Opinion Score = q20+q22+q23+(6-q21)+(6-q24) 1) <15 (n = 62)	2) 15-17 (n = 105)	3) 18+ (n = 63)	p Value#
1. For how many years have you been an IRB chair?	n	62	105	63	0.41^^^
	5+	38 (61%)	55 (52%)	34 (54%)	
	<5	24 (39%)	50 (48%)	29 (46%)	
31-37. Number of correct answers in q31 q32 q34 q35 q36 q37	n	61	101	62	0.09^^^
	1) <3	31 (51%)	47 (47%)	22 (35%)	
	2) 3+	30 (49%)	54 (53%)	40 (65%)	
3. How familiar are you with Certificates of Confidentiality? (Recoded)	n	62	105	63	0.53^^^
	1=More familiar	34 (55%)	34 (32%)	38 (60%)	
	2=Less familiar	28 (45%)	71 (68%)	25 (40%)	
30. Have any studies at your institution ever received a legal demand (e.g., a subpoena, a court order, or other formal request) to disclose identifiable research data?	n	41	60	44	0.06^^^
	No	36 (88%)	52 (87%)	32 (73%)	
	Yes	5 (12%)	8 (13%)	12 (27%)	

Q26 [AMONG Q25 = Greater than minimal risk (n=190)] -Would your IRB approve this research without a Certificate of Confidentiality?

Measure	Statistic/ Category	26. Would your IRB approve this research without a Certificate? No (n = 88)	Yes (n = 84)	p Value#
1. For how many years have you been an IRB chair?	n	88	84	0.23^
	5+	53 (60%)	43 (51%)	
	<5	35 (40%)	41 (49%)	
31-37. Number of correct answers in q31 q32 q34 q35 q36 q37	n	88	84	0.010^
	1) <3	27 (31%)	42 (50%)	
	2) 3+	61 (69%)	42 (50%)	
3. How familiar are you with Certificates of Confidentiality? (Recoded)	n	88	84	0.03^
	1=More familiar	49 (56%)	33 (39%)	
	2=Less familiar	39 (44%)	51 (61%)	
20-24. Composite opinion score = q20+q22+q23+(6-q21)+(6-q24)	n	84	82	0.92^^^
	1) <15	23 (27%)	21 (26%)	
	2) 15-17	35 (42%)	38 (46%)	
	3) 18+	26 (31%)	23 (28%)	
30. Have any studies at your institution ever received a legal demand (e.g., a subpoena, a court order, or other formal request) to disclose identifiable research data?	n	57	47	0.42^
	No	45 (79%)	40 (85%)	
	Yes	12 (21%)	7 (15%)	

27. [AMONG Q25 = Greater than minimal risk (n=190)] If the Principal Investigator does obtain a Certificate how would you categorize the level of risk involved in this study?

Measure	Statistic/ Category	27. If the PI does obtain a Certificate how would you categorize the level of risk involved in this study? Greater than minimal risk (n = 135)	No greater than minimal risk (n = 50)	p Value#
1. For how many years have you been an IRB chair?	n	135	50	0.78^
	5+	76 (56%)	27 (54%)	
	<5	59 (44%)	23 (46%)	
31-37. Number of correct answers in q31 q32 q34 q35 q36 q37	n	135	50	0.98^
	1) <3	57 (42%)	21 (42%)	
	2) 3+	78 (58%)	29 (58%)	
3. How familiar are you with Certificates of Confidentiality? (Recoded)	n	135	50	0.18^
	1=More familiar	58 (43%)	27 (54%)	
	2=Less familiar	77 (57%)	23 (46%)	
20-24. Composite opinion score = q20+q22+q23+(6-q21)+(6-q24)	n	133	46	0.02^^^
	1) <15	41 (31%)	6 (13%)	
	2) 15-17	56 (42%)	22 (48%)	
	3) 18+	36 (27%)	18 (39%)	
30. Have any studies at your institution ever received a legal demand (e.g., a subpoena, a court order, or other formal request) to disclose identifiable research data?	n	85	28	0.86^
	No	71 (84%)	23 (82%)	
	Yes	14 (16%)	5 (18%)	

28. [AMONG Q25 = Greater than minimal risk (n=190)] If the Principal Investigator obtains a Certificate how would you characterize the change in protection provided to research participants in this study?

Measure	Statistic/ Category	28. If the PI obtains a Certificate how would you characterize the change in protection provided to participants in this study? 1) Decreases protection (greatly or slightly) (n = 14)	2) No difference in protection (n = 12)	3) Increases protection (greatly or slightly) (n = 164)	p Value#
1. For how many years have you been an IRB chair?	n	14	12	164	0.77^^^
	5+	8 (57%)	5 (42%)	92 (56%)	
	<5	6 (43%)	7 (58%)	72 (44%)	
31-37. Number of correct answers in q31 q32 q34 q35 q36 q37	n	14	12	164	0.39^^^
	1) <3	4 (29%)	6 (50%)	72 (44%)	
	2) 3+	10 (71%)	6 (50%)	92 (56%)	
3. How familiar are you with Certificates of Confidentiality? (Recoded)	n	14	12	164	0.42^^^
	1=More familiar	8 (57%)	5 (42%)	72 (44%)	
	2=Less familiar	6 (43%)	7 (58%)	92 (56%)	
20-24. Composite opinion score = q20+q22+q23+(6-q21)+(6-q24)	n	13	12	158	0.35^^
	1) <15	3 (23%)	8 (67%)	37 (23%)	
	2) 15-17	6 (46%)	2 (17%)	73 (46%)	
	3) 18+	4 (31%)	2 (17%)	48 (30%)	
30. Have any studies at your institution ever received a legal demand (e.g., a subpoena, a court order, or other formal request) to disclose identifiable research data?	n	12	7	97	0.89^^^
	No	10 (83%)	6 (86%)	80 (82%)	
	Yes	2 (17%)	1 (14%)	17 (18%)	

^	Pearson's chi-square test
^^	Cochran-Mantel-Haenszel test on nonzero correlation
^^^	Cochran-Mantel-Haenszel test on row mean scores difference
#	Wilcoxon rank sum/Kruskal-Wallis test was used for all continuous row variables

Source: /ccge/Projects/CofC/program/FinalRun.sas; Date: 28MAR12
