# Supplementary material for: Institutional Review Boards’ Use and Understanding of Certificates of Confidentiality
Source: PLoS One. 2012 Sep 4;7(9):e44050. doi: 10.1371/journal.pone.0044050 (PMC3433491; doi:10.1371/journal.pone.0044050)
Supplement: Appendix S2 — Additional Sample Quotes. (PDF) [file pone.0044050.s002.pdf]

APPENDIX S2: ADDITIONAL SAMPLE QUOTES

(Beskow LM, et al. IRBs' use and understanding of Certificates of Confidentiality)

Use of Certificates of Confidentiality

Certificates exclusively to protect information about illegal activity

- "[A Certificate] protects behaviors which, if they were made public or made available to legal authorities, might result in the research subjects being arrested or at least investigated." (H43803)
- "If you started requesting it for studies that are just collecting gene specimens, I think some [NIH] institute's offices when you submit it will say, What is that? It's not commonly used for that… I don't think the National Heart, Lung has much experience with Certificates of Confidentiality. Smoking is not an illegal behavior." (L43632)

Certificates mostly to protect information about illegal activity

- "If confidentiality was breached because an attorney subpoenaed your files, I might get embarrassed, but nothing I'm saying here is illegal. Whereas if I am doing research about cocaine/crack use, much of what people are telling me constitutes illegal criminal activity. And so if the DA subpoenaed my research files, these people are under real risk. While I won't get into whether they should or shouldn't go to jail, they certainly shouldn't do so as part of agreeing to participate in my research…" (L43632)
- "There are some cases that seem extremely important to get a confidentially certificate… if we're collecting information about illegal conduct, and that could be drug trafficking, that could be immigration, it could be any number of issues where it's prima facie illegal to do that." (HD43839)

Assessing the need for Certificate: Foreseeable risk of litigation

- "If there wasn't any risk of subpoena, then we wouldn't [require a Certificate]. Regardless of the data we wouldn't insist on the thing. So if there's no legal issue it's just not relevant." (L43559)
- "I mean clearly there is sensitive and potentially damaging information and if … that information is going to be out there and there is a substantive risk that someone might be interested in obtaining this information through legal means, then we would make that recommendation … to obtain a Certificate." (LD43814)
- "We perceive [Certificates] primarily as a way of reducing risk in research where there is a potential threat of forced release through subpoena… It would just depend on whether this is an area where there is a reasonable—I mean based on the kind of suits that we were aware were being brought—whether there was a reasonable expectation that based on litigation history, whether a Certificate might be something we would consider." (L43559)

Assessing the need for Certificate: Realistic threat of serious harm

- "It has to be a serious matter and not just a vague possibility that it would be disclosed. Other items are in there for confidentiality protection—separating the names and things. So you've already got a fair amount of protection there." (H43577)
- "Our board, they have the perception that these are extremely spectacular protocols that should require a Certificate of Confidentiality." (L43838)
- "If it's illegal conduct, then it is much more likely that this research will become known or relevant to an actual court proceeding, in which case there may actually be a subpoena that needs to be defended against. In terms of the financial standing, there are potentially cases where there could be a 20-year ruin to somebody's life. And similarly with the social stigmatization or even the sexual attitudes or preferences. So those are all cases where what we would look at is again, the magnitude of the harm along multiple dimensions and the likelihood that a Certificate would be helpful in reducing the chances of a confidentiality breach." (M43623)
- "Generally speaking, I think Certificates are useful. I would hate to see them done away with... I just think quite frankly, in the vast majority of circumstances, the vulnerability that subjects might have when they disclose confidential information … tends to be kind of exaggerated in the minds of some researchers and some board members… Some of the scenarios, at least in the past, that some board members have brought up about somehow a county prosecutor or somebody is going to try to subpoena a researcher's records to find out who is dealing drugs in the community or whatever, I just don't think … those scenarios are very realistic. I understand that all that has to happen is for that kind of thing to happen once, word gets around, and that's the reason why the Certificates exist. It's just that I think a lot of the scenarios on which those rationales are based are pretty low probability events." (H43803)
- "The prime thing that we look at really is on illegal conduct, or some sort of litigation threat. And if that's there, we're going to move quickly to say, 'Well you should have a Certificate.' On the broader question about employability sensitivity, that's tricky because it has to be really serious… Certificates are not given lightly. When you think about it, the government is saying, 'We aren't going to come after you for something.' And that's rare in law. So it's a big deal to actually get one of these." (H43577)

Assessing the need for Certificate: Availability of information elsewhere

- "Anything that would be in your medical record, why would we require a Certificate of Confidentiality when you can subpoena the medical record?" (L43632)
- "From my point of view, any information that—even though I'm doing it more systematically [for research purposes]—will be part of what I could do as part of the medical record, I'm not required to have a Certificate. Meaning, I don't administer formal depression screening in clinical practice, but if I think somebody is depressed, I note it down in the chart. So that's the example I would give, that I would never think of requiring a Certificate for that." (L43632)

- “We think it’s intended to protect subjects when we’re asking them to disclose behavior that puts them at risk were it known. And that would not be in the medical record. You have cancer and they make you uninsurable, it may cost you your job, but that’s already in your medical record. Then why do I need Certificate for the poor researcher? Any insurance company can get the medical record.” (L43632)

Certificates and genetic information: Association with illegal activity

- “I could see how when you got into genetic research, into the causes let’s say of criminal behavior, that eventually [Certificates] might be utilized.” (H43803)
- “I think where the genetic tests come in is whether the genetic data could at some point be used in a legal proceeding. That’s a little hard to know when somebody is doing a study that’s not particularly related to any sensitive area, but nonetheless, you’ve got genetic data that could link somebody to a crime and be used for other purposes. I think some of our boards would probably say we should require one here and some would not.” (L43699)

Certificates and genetic information: Association with other sensitive data

- “We have a large group on campus who do research … looking for the overlay between environmental and genetic variables. They do ask about quite sensitive activities and some that would be illegal for an adolescent to admit to or, it wouldn’t be illegal but it wouldn’t look so good if a parent admitted that their adolescent was involved in these behaviors. But it’s the overlay between the two—we worry about the mix of genetic markers for a particular racial and/or ethnic group and then what that might do in their lives as individuals, or as a group even. So then we recommend the Certificate.” (MD43786)

Certificates and genetic information: Genetic information is not sensitive

- “We’ve also found that some [NIH institutes have] required Certificates for sort of ho-hum genetic stuff that we find doesn’t really warrant it. And we end up just caving to that, but it seems silly… When we recommend or require a Certificate, we mean business at our IRB. It’s not some silly little genetic thingy; it’s a real deal. And so that’s what we save it for.” (L43636)
- “We do not routinely recommend [Certificates] for genetic or banking studies and find it surprising that some groups do. We feel this overblows the risk of those studies as we are not familiar with legal harm that has come of such data collection. We feel this rather “dilutes” their appeal/meaning. (L43636)

Certificates and genetic information: Existing genetic privacy laws

- “We have a genetic privacy law here in [state] that we think covers genetic research specifically.” (HD43857)

Certificates and genetic information: Accessibility of widely-shared genetic data

- "In the case of repositories that are designed to share the data on a widespread basis, and especially repositories that deal with genome-wide association studies, I think that the subject is assuming a very high probability of confidentiality not being able to be protected. So I don't think we would [recommend a Certificate] in a case like that." (MD43561)

Opinions about Certificates of Confidentiality

Protection of identifiable research data: Nearly absolute protection

- "The researcher would be free of the obligation to deliver data in a lawsuit." (MD43786)
- "Well, nearly absolute… There are a few things where there are other laws that trump the Certificate. It might vary state by state—but otherwise, things like divorce proceedings or credit investigations or things like that can't override the Certificate." (HD44066)

Protection of identifiable research data: Strong deterrent to access

- "Having the Certificate conveys to potential lawyers or litigants that there's an uphill battle here and it's going to get expensive, and they shouldn't waste their time. We have an attorney on our board that concurs with that position, which reassured the board itself that these were pretty interesting kinds of documents. So we don't say absolute, but it's pretty close." (H44037)
- "The perception among lawyers is if there's a Certificate, find another way to get information; don't try a subpoena. So it has a huge deterrent function." (H44037)
- "We think that if a lawyer goes to court, say to defend against a subpoena or some other request, the lawyer is better off if he's got the Certificate in hand along with a nice, strong consent form that has provided participants with the presumption of privacy of their information." (HD43839)

Protection of identifiable research data: Barrier to access

- "At best [Certificates] throw up an additional barrier that may add to the time and expense required to obtain confidential information. That may encourage some compromise or negotiation between parties but ultimately I don't think it's enough to prevent the release of confidential information in practice." (L43559)
- "I suspect that … if there was a legal request for the documents, that some kind of deal would be worked out because we quite honestly don't believe that most institutions, forced with a subpoena for this information, are going to spend the money to go through the courts and to try to block the subpoena whether a Certificate is there or not. I think the Certificate might give people a little bit more room for negotiation and might slow down the release, but I'm not sure that

ultimately it would prevent the release… So I'm skeptical that in practice it provides much benefit." (L43559)

Protection of identifiable research data: Limits to protection

- "It doesn't protect against the investigator getting a little loose with the information and it doesn't protect against the individual wanting to tell everybody what they told the investigator. It just keeps the third parties out of the mix." (H44037)
- "[A Certificate] obviously doesn't affect the risk of disclosure, for example, of somebody seeing a participant walking into a study site. It doesn't protect against disclosure that happens because somebody erroneously had the information on a portable hard drive that they left in a coffee shop. I think the Certificate protects against a kind of worst case scenario. There are a number of situations in which we don't see that there's much need or reason because the possibility of a breach that actually would have been stopped by the Certificate is quite small." (M43623)
- "I think there's still a risk of the information that's provided, human error happens. We've had a number of laptop thefts. I mean [a Certificate is] not a panacea. It's not magic. It doesn't eliminate the risk of having potentially damaging information. I think it marginally alters the risk of collecting sensitive data." (L43636)
- "I think Certificates are frequently misunderstood by people as essentially giving a kind of 'absolutely nothing that is ever said will ever be known to anyone else and there's no possible way for this information ever to get out.' … I mean it's not a cone of silence. It's not the case that this information is somehow not recorded or nobody knows or nobody could ever find out. And I think that that can lead to a false sense of security on the part of participants. I think also it can lead to issues in terms of people being willing to take on risks that they might otherwise not have done. One worry that we always have when we're reviewing protocols where people are talking about getting a Certificate is making it clear in the consent forms and the consenting processes that there are still risks. Invariably in these studies there are still risks that the Certificate is not relevant to… It's sort of these findings that when people start wearing helmets, often there was a period where they were injured more frequently because they would take chances they otherwise wouldn't have taken. I think Certificates can play that role sometimes, unfortunately." (M43623)

Protection of identifiable research data: Institutional willingness to fight disclosure

- "The relevance and meaning of Certificates also depends to a potentially considerable degree on the willingness of the institution to fight administratively, legally and politically against disclosure of collected information, with or without the existence of a Certificate." (H43816)
- "I think a Certificate adds a little bit of an extra legal barrier, but I think if a lawyer really wanted to get the data, they'd push for it and they'd probably get it. I'm just really skeptical that a Certificate really reduces the risk that a subpoena will be successful… I don't think a Certificate means anything unless the institution where

the research is being done is willing to say, ‘We’re going to invest money in lawyers to make the Certificate stick.’ I just don’t … I think they’re going to deal. They’re going to make a deal with the lawyers on the other side.” (L43559)

- “I suspect that institutions who apply for Certificates, that if there was a legal request for the documents, that some kind of deal would be worked out because we quite honestly don’t believe that most institutions … are going to spend the money to go through the courts and to try to block the subpoena, whether a Certificate is there or not. I think the Certificate might give people a little bit more room for negotiation and might slow down the release, but I’m not sure that ultimately it would prevent the release.” (L43559)

Protection of identifiable research data: Importance of standard confidentiality measures

- “Quite simply, we look at the information collected. We look at how it’s labeled or stored, linked to an individual or not, and who has access to it, where it might be stored, how long it’s stored and that sort of thing. I mean we expect a certain level for all studies. We advise coding of information rather than using directly identifiable information. We ask people with whom data would be shared, internal collaborators, external collaborators, and how it would be labeled in that setting. And we sort of put all this together and decide, does this past muster? Might special protections be needed?” (L43636)

Protection of identifiable research data: Extra layer of protection

- “As is the case with most or virtually all IRBs, our members see the Certificate as providing you with kind of an extra layer of protection, particularly in projects that are collecting data which are super sensitive, so to speak.” (H43803).
- “One of the pros is that if [a Certificate is] in place, it tends to convey an added layer of protection for subjects. It also tends to remind investigators about the need for confidentiality.” (H44037)
- “We have viewed Certificates as providing an extra layer of protection from subpoenas and other attempts to obtain those records in situations where you are getting information, not that’s embarrassing, but that actively puts people at risk.” (L43632)
- “I’m not sure that we find [Certificates] to be a guarantee. We find it a small extra measure. We find it can be reassuring, again when we’re talking about things like illegal behaviors or perhaps motor vehicle accidents, substance use, STDs, HIV, sexual behaviors or deviancies or whatever you want to call it. I think we view it as a small extra potential protection … mostly were the information to be requested by some legal proceeding is presumed to be disadvantageous to the subject.” (L43636)
- “One advantage is [a Certificate] gives you an added layer of protection against being compelled to reveal confidential information, particularly confidential information that may put somebody at risk. And presumably that has benefits to both the participant in the research and probably the research team.” (L43699)

- “In my experience, the Certificate provides an additional … layer is probably not the best word … but an additional component to protect the confidentiality of research data.” (L43768)
- “I would consider their role as a sort of an extra means to protect researchers, to insure confidentiality, and then if it would come into question, then the data could be protected through the use or presence of a Certificate.” (L43768)
- “My understanding of what [Certificates] are is to add an extra layer of protection when there are risks of confidentiality issues. One of the things that we consider in protection of human subjects is issues associated with confidentiality and this sort of acts as an extra additional protection … over and above what the IRB does at a local level.” (M43621)
- “I think they’re good. Being social behavioral, we have a lot of researchers who want to do things that have risks associated with confidentiality, so they’re really good for those researchers to be made aware of and have that extra protection.” (M43621)

Protection of identifiable research data: Importance of standard measures

- “Quite simply, we look at the information collected. We look at how it’s labeled or stored, linked to an individual or not, and who has access to it, where it might be stored, how long it’s stored and that sort of thing. Kind of put together those pieces of information and decide whether special confidentiality precautions are needed or not. I mean we expect a certain level for all studies. We advise coding of information rather than using directly identifiable information. We ask people with whom data would be shared, internal collaborators, external collaborators, and how it would be labeled in that setting. We sort of put all this together and decide, does this pass muster? Might special protections be needed? (L43636)
- “A lot of the IRB’s work comes down to information security, how to lock things down, how to keep it encrypted or stripped out, make sure that identifiers are either not collected or their use and collection is very limited, or the time period is very limited while they’re retained. Where there’s a risk of a subpoena, we’d say, ‘Well, you know the best way to prevent that risk is to kill the identifiers.’” (L43559)

Scope of protection: Had been or would be upheld in court

- “Well, we have two attorneys with my institution and they’ve both indicated that there’s never been a court case that said a Certificate would not be honored. In other words, it’s never been overturned in a court. So that’s pretty strong evidence that the protection will hold up.” (H43577)
- “The major advantage is that [a Certificate] gives the researcher a sense of confidence that, in fact, the confidentiality they’ve guaranteed to the research participant could be upheld even upon certainly a legal redress. A second [advantage] might be that with the Certificate, the researcher may feel more confident to ask increasingly or increased sensitive questions or questions about sensitive material.” (MD43786)

- "We have read some information and my final impression has been that the Certificate indeed has protected participants and principal investigators in terms of not making him provide information tied from any other legal implications which are not covered by the Certificate." (L43838)

Scope of protection: Largely untested in court

- "I mean the pros on the face of it are that [a Certificate] reduces risk, that it will provide some kind of barrier against the subpoena. I'm not sure whether that's true in practice, largely because it's untested, or there seems to be very little testing in the courts." (L43559)
- "I think a disadvantage is that they're not really tested legally. We have a few cases, but we don't really know if they would hold up. We've used them a few times here, but it's never been pressed to the max." (HD43857)
- "To my knowledge at least, at present the Certificate has not been clearly tested in court. So I don't think we really know that it will be upheld by the judiciary." (LD43588)
- "My understanding [is] that this hasn't been tested. Now maybe it has and I'm not aware of it, but that's the information that I'm operating under. And in the case that I described to you, our legal counsel was involved, and that was the same assumption, the same understanding that we had at that time, that this has not been tested. So I could not agree with the statement that this has been upheld in court cases." (L43768)

Scope of protection: Skeptical that would be upheld in court

- "I'm personally unaware of specific situations in which they've been tested. Rumor circulates among some investigators in our institution that if information is really subpoenaed, that they may not hold up." (L43636)
- "There have not been a lot of legal challenges and, therefore, it is really in my mind uncertain the level of protection that [Certificates] actually afford. And obviously, in the unhappy circumstance that one would be successfully challenged, there is a huge con to both the subjects and the researchers of having this disclosure happen." (L44006)
- "The pros are that [a Certificate] makes everyone feel better, although it is unclear how much better it should make you feel… They have not really been subjected to legal challenges." (L43632)
- "[In study involving videotape of driving behavior] there were questions about the extent to which it could protect or allow the researchers to resist the request for research data. It was our understanding from our legal counsel that if there were a legal action based on some driving event and the attorneys for either side were aware that this record existed, the Certificate may not protect the researcher entirely from turning over the video." (L43768)

- “I think it’s not really clear, because of the lack of caseload, how effective [Certificates] really are… I think unfortunately, IRBs and researchers tend to think that they are pretty much bulletproof.” (L44006)
- “I think there is probably a huge sort of either misperception or a huge gap between perhaps what NIH intends for them to protect and what most researchers and institutions that are conducting social behavioral research believe that the Certificate will cover. It worries me a little bit that a project gets them and the IRB is happy about that, and the PI thinks they’re covered, and the fact that we really don’t know, because there haven’t been enough challenges, we don’t really know how well they hold up and what protections they afford.” (L44006)

Researchers’ sense of security: Certificates’ effectiveness

- “I don’t think they’re getting a false sense of security because [a Certificate] is pretty secure.” (H43577)
- “A problem with Certificates [is] this potential false sense of security, if they indeed are not as foolproof as I think most researchers and IRBs perceive them to be.” (L44006)

Researchers’ sense of security: Certificates would lessen focus on other protections

- “They can give investigators a false sense of security that might in some cases lead to less stringent procedures, practices and vigilance in the provision of more “ordinary” protective measures.” (H43816)
- “There might be a temptation not to employ very diligent safeguards of the data. The Certificate does not replace that. It’s in addition to all the things that one is supposed to do. So I suppose that there could be kind of this artificial sense of, ‘oh, I might not have to do other things.’” (L43768)

Researchers’ sense of security: Need for education

- “I remember a case years ago where … everybody got sued. And the researcher was, ‘My data!’ I mean sometimes researchers are incredibly naïve. They said, ‘They can’t, I’m a researcher. I’m protected. I shouldn’t have to release my research data!’ I mean [laughter] you’re kidding me! When the lawyers come after you, you’ll do whatever the court’s telling you to do or go to prison… I think there should be a lot better education of researchers that they’re at risk.” (L43559)

Promotion of Research Participation: Reassuring prospective participants

- “Many people, when sensitive topics are being discussed, are understandably quite hesitant to speak about their own experiences or the experiences of those that they know or have witnessed. I think Certificates can provide people with a level of comfort that enables them to be willing to participate in research in the first place.” (M43623)
- “I’ve heard investigators say that they thought [a Certificate] was very helpful—particularly in studies involving criminal conduct, illegal behavior, illegal

immigration, and various disease categories—that the subjects felt reassured and liked to see that language. And they felt that it was easier to recruit subjects as a result of that. More important, I've heard from some investigators that it was easier to retain subjects, particularly in repeat visit studies. They felt more comfortable and more reassured about things being held in strict confidence." (H44037)

- "In some research where enrollment may be difficult because of the nature of the disclosures the participants are making, a Certificate provides extra assurance to them that it's okay to participate and that the risk of that information getting out is going to be decreased." (HD43857)
- "[Certificates] came in historically because all the research we do in health is asking pretty sensitive questions, which if the answers got out could actually get either in legal trouble or could certainly cause a lot of serious problems in terms of employability, reputation and so on. In order to get them to participate, [a Certificate] does give a certain amount of protection, and otherwise they might not be willing to participate if they didn't get it." (H43577)
- "Subjects who see that there is this extra layer of protection structured into the design are more likely to participate, particularly if the study calls for a lot of disclosure of sensitive information." (H44037)
- "Certificates help the researchers protect the information that they're given by the subjects, especially when that information is about legal behaviors or things that could be harmful to the participant if they were known. So it really is a tool for the researcher to increase participation in topics that would otherwise perhaps not get as good of participation rates." (HD43659)
- "Sometimes researchers think, but again we don't have data, [that a Certificate] improves their ability to get participation in sensitive studies." (HD43839)
- "[For] research where people might put themselves at some legal risk of the information being disclosed, [a Certificate] allows that research to go on without requiring the subjects to take on that additional risk. It's an important tool." (L44006)

Promotion of Research Participation: Explaining the limitations

- "I'm just really skeptical that [Certificates] do very much. From an IRB point of view we tend to say, 'Well if they don't cost very much and if they're easy to get, why not get them because it might do something, we just don't know.' But a downside to that is that you've got to explain what we think the limitations are to human subjects, and that might confuse them or it might mislead them. So it's really difficult to have strong feelings that the net benefits are there." (L43559)
- "We've heard indirectly that participants typically seem, in the researcher's view, overly reassured about what the Certificate does. And so they report having to spend a fair amount of extra time … explaining all the things that are in fact not covered by the Certificate." (M43623)

Promotion of Data Quality: Reassuring prospective participants

- "If you're asking people about a whole history of criminal behaviors and whether they're continuing to use illegal substances and things like that, they're not going to share that information in many situations unless they're assured, or at least the probabilities are decreased, that this information is going to harm them. So it seems to me that [a Certificate is] really an important tool." (HD43659)
- "On research with high risk and sensitivity, participants' willingness to provide … valid data about themselves is going to be influenced by how confidential they think it's going to be kept. If you have a Certificate, that not only reminds them that there's some protection against third party intrusion, but that the whole concept of confidentiality is being taken seriously by the research team… Now whether they tell you the absolute truth or not is kind of independent of the Certificate—if they're going to make stuff up, it doesn't matter whether you have a Certificate. But they're more likely to tell you what's actually true if they think the information will be held secret." (H44037)
- "I think it can be reassuring to subjects. It could allow them to provide information that they might otherwise be hesitant to provide." (L43636)
- "I think they're useful and really actually help researchers—even though there's more paperwork involved—to increase the likelihood of being able to communicate with the kinds of participants they want to and get accurate and honest information from them. So I think that they're a real advantage in much of the research." (HD43659)
- "I think there's actually a scientific benefit to Certificates that is often not talked about, which is that they enable us to quite frankly have a bit more confidence in data. Data about sensitive topics is notoriously subject to lots and lots of problems, whether it be sample selection bias, reporting errors, compliance problems. And I think Certificates can do quite a lot in terms of trying to help us get cleaner data because [many of these problems] actually can be mitigated by the presence and sort of broadcast advertising of the existence of a Certificate—when people actually understand what [Certificates] cover, which I think frequently they don't." (M43623)

Promotion of Data Quality: Certificates not needed

- "In my experience, subjects are often willing to disclose rather surprising things without having to go through all this. Even asking them a tame question, they may disclose something that it really could come back to haunt them." (H43577)

Promotion of Data Quality: Falsely reassuring prospective participants

- "I mean it's not a cone of silence. It's not the case that this information is somehow not recorded or nobody knows or nobody could ever find out. I think that can lead to a false sense of security on the part of participants. I think also it can lead to issues in terms of people being willing to take on risks that they might otherwise not have done." (M43623)

- “I would say the disadvantages of them are I think they may provide a false sense of security. That’s my own kind of cynical viewpoint. Perhaps people think that there’s some super-special limitation or misunderstand who might have access to the information because it has this Certificate… I also think people do not understand that some of the potentially most damaging information that they might provide, for example, things about abuse, things of that nature, we still have state reporting requirements there that are not obviated by a Certificate of confidentiality.” (L43636)
- “A disadvantage in theory would be that it provides a misleading perception to participants about protection that they might have.” (LD43814)

Promotion of Data Quality: Researcher-participant trust is most important

- “I really believe that your relationship with the participant enhances their trust and what they’re going to say. Unfortunately, the more documents people see does not necessarily make them feel any better… Anything more from the government, I don’t think helps that in any way.” (L44010)
- “If you don’t trust the man, you ain’t giving him the information with or without the Certificate.” (L43632)

The Effect of Certificates on Assessments of Research Risk

Certificates could lower the level of risk involved

- “If that was the only thing—that we felt like it was information that in and of itself wasn’t necessarily damaging, but in certain circumstances this compelled disclosure could be damaging—then yeah, using a Certificate would I think put it back to minimal risk, if that was the only factor.” (HD44066)
- “We had a study that was clearly minimal risk, but it did ask questions about I believe it was drug use. The decision was made that it was only eligible for expedited review if they got a Certificate. The team didn’t want to get one. And we decided that it increased the potential risk enough that it was no longer eligible for expedited review, and would have to go to the full board if they didn’t want to do that. So we basically gave them a choice. They could get a Certificate and have it be expedited, or they could bring it to the full board and see if the board would require it.” (L43699)

Certificates help mitigate risk but do not change risk categorization

- “We don’t knock something down from a greater than minimal risk to minimal risk with the presence of a Certificate. I know some do that, but we think that it is a tool to manage risk. We wouldn’t change our review category based on the fact that a Certificate is in place.” (HD43857)

- “If we had a study which we didn’t feel we could approve because the risk was too high, and we asked the investigator to get a Certificate, that may be the factor that would say, okay, with that protection we could approve it. We would not tend to lower something we considered full committee to expedite it based on the Certificate. I think we’d say if it merits a Certificate, it’s probably more than minimal risk.” (MD43561)
- “I think most of the projects for our IRB where a Certificate is involved are typically full board or expedited reviews. But I think most of them are full board reviews, and attaching a Certificate to the whole process, I don’t recall a protocol in which because a Certificate was there it pushed it back down let’s say to expedited. I can see the logic for that though. I just don’t recall any cases in which that happened with our IRB.” (H43803)
- “If there’s risk that requires a Certificate then we require having the Certificate. The Certificate comes after the risk determination. Yeah, if we require a Certificate of Confidentiality it’s going to stay more than minimal risk. The key here is I and our board members want to be sure that we’re managing the risk properly and that the PI is deeply involved in managing the risk. And the Certificate is part of that management scheme. But just having one doesn’t change the risk to minimal.” (H44037)
- “I don’t think that the IRB will reduce the level of risk assessment, but they will be more than willing to approve [a more than minimal risk] study with a Certificate.” (L43838)

Plans to obtain a Certificate: Effect on the IRB

- “If this were the only risk, it certainly would allow us to possibly consider the project as no more than minimal risk, if the investigator has shown how they will protect the confidentiality of the subjects.” (LD43588)
- “In some ways, [a Certificate is a] flag that the researcher thinks this is potentially a very problematic study. I feel sort of guilty saying that … this plays a role, but it does. I mean if the researcher says, I think I might need this, then it certainly gets very closely scrutinized, which is not to say that other studies do not, but I think it certainly is a signal to us as an IRB.” (M43623)
