# Supplementary material for: Institutional Review Boards’ Use and Understanding of Certificates of Confidentiality
Source: PLoS One. 2012 Sep 4;7(9):e44050. doi: 10.1371/journal.pone.0044050 (PMC3433491; doi:10.1371/journal.pone.0044050)
Supplement: Box S1 — Hypothetical Research Scenario. (PDF) [file pone.0044050.s003.pdf]

Investigators plan to evaluate a needle exchange program (NEP) by studying how it affects drug users' injection-related risk behaviors. Participants in the study will be interviewed at baseline and 6 month intervals. Interviews will assess five injection related risk behaviors: receptive needle sharing, lending needles, sharing paraphernalia, needle reuse, and cleaning of needles. Injection drug users will be recruited from the NEP, and a control group of injection drug users will be recruited from a nearby town that does not have a NEP. Data collected will be linked to identifying information in order to compare interview responses longitudinally. In addition to employing standard physical and electronic security measures, identifying information will be removed from interview data and replaced with a code. The list that links the code to identifiable information will be kept in a secure location, accessible only by select study personnel. (Adapted from Huo & Ouellet 2007)
